# Controlling hepatitis C in Rwanda: a framework for a national response

**DOI:** 10.2471/BLT.16.183772

**Published:** 2017-11-27

**Authors:** Aimable Mbituyumuremyi, Jennifer Ilo Van Nuil, Jeanne Umuhire, Jules Mugabo, Mutagoma Mwumvaneza, Jean Damascene Makuza, Justine Umutesi, Sabin Nsanzimana, Neil Gupta

**Affiliations:** a Rwanda Biomedical Center, Kigali, Rwanda.; b Partners in Health, Inshuti Mu Buzima, KG 9 Avenue, Plot No. 46, Kigali, Rwanda.; c Clinton Health Access Initiative, Kacyiru, Rwanda.; d World Health Organization, Kigali, Rwanda.; e Division of Global Health Equity, Brigham and Women’s Hospital, Boston, United States of America.

## Abstract

With the introduction of direct-acting antiviral drugs, treatment of hepatitis C is both highly effective and tolerable. Access to treatment for patients, however, remains limited in low- and middle-income countries due to the lack of supportive health infrastructure and the high cost of treatment. Poorer countries are being encouraged by international bodies to organize public health responses that would facilitate the roll-out of care and treatment on a national scale. Yet few countries have documented formal plans and policies. Here, we outline the approach taken in Rwanda to a public health framework for hepatitis C control and care within the World Health Organization hepatitis health sector strategy. This includes the development and implementation of policies and programmes, prevention efforts, screening capacity, treatment services and strategic information systems. We highlight key successes by the national programme for the control and management of hepatitis C: establishment of national governance and planning; development of diagnostic capacity; approval and introduction of direct-acting antiviral treatments; training of key personnel; generation of political will and leadership; and fostering of key strategic partnerships. Existing challenges and next steps for the programme include developing a detailed monitoring and evaluation framework and tools for monitoring of viral hepatitis. The government needs to further decentralize care and integrate hepatitis C management into routine clinical services to provide better access to diagnosis and treatment for patients. Introducing rapid diagnostic tests to public health-care facilities would help to increase case-finding. Increased public and private financing is essential to support care and treatment services.

## Résumé

Grâce à l'introduction d'antiviraux à action directe, le traitement de l'hépatite C est à la fois très efficace et bien toléré. Néanmoins, l'accès des patients au traitement demeure limité dans les pays à revenu faible et intermédiaire en raison du manque d'infrastructures sanitaires de soutien et du coût élevé du traitement. Les pays pauvres sont encouragés par des organismes internationaux à élaborer des mesures de santé publique qui faciliteraient la mise en place de soins et de traitements à l'échelle nationale. Peu de pays ont cependant établi des politiques et des plans officiels. Dans cet article, nous présentons l'approche adoptée au Rwanda à l'égard d'un cadre de santé publique pour le contrôle de l'hépatite C et les soins qui lui sont associés dans le contexte de la stratégie du secteur de la santé contre l’hépatite de l'Organisation mondiale de la Santé. Cela inclut le développement et la mise en œuvre de politiques et de programmes, d'efforts de prévention, de capacités de dépistage, de services de traitement et de systèmes d'information stratégiques. Nous mettons en avant les principaux succès du programme national pour le contrôle et la gestion de l'hépatite C: l'établissement d'une gouvernance et d'une planification nationales; le renforcement des capacités de diagnostic; l'approbation et l'introduction de traitements antiviraux à action directe; la formation de personnel d'encadrement; le développement d'une volonté et d'un leadership politiques; et la promotion de partenariats stratégiques clés. Les enjeux actuels et les prochaines étapes du programme incluent l'élaboration d'un cadre détaillé de suivi et d'évaluation, ainsi que des outils pour le suivi de l'hépatite virale. Le gouvernement doit favoriser la décentralisation des soins et intégrer la gestion de l'hépatite C aux services cliniques courants afin de fournir aux patients un meilleur accès au diagnostic et au traitement. L'utilisation de tests de diagnostic rapide dans les établissements publics de santé permettrait d'améliorer le dépistage. Il est essentiel d'augmenter les financements publics et privés pour soutenir les services de soins et de traitement.

## Resumen

Con la introducción de los fármacos antivíricos de acción directa, el tratamiento de la hepatitis C es altamente eficaz y tolerable. Sin embargo, el acceso al tratamiento por parte de los pacientes sigue siendo limitado en los países de ingresos medios y bajos, debido a la falta de infraestructuras sanitarias de apoyo y a los altos costos del tratamiento. Los organismos internacionales alientan a los países más pobres a organizar respuestas de salud pública que podrían facilitar la puesta en marcha de atención y tratamiento a escala nacional. Sin embargo, son pocos los países que han documentado planes y políticas formales. En el presente estudio, esbozamos el enfoque adoptado en Rwanda para un marco de salud pública para el control y la atención de la hepatitis C dentro de la estrategia del sector de la salud contra la hepatitis de la Organización Mundial de la Salud. Este incluye la elaboración y aplicación de políticas y programas, medidas preventivas, capacidad de cribado, servicios de tratamiento y sistemas de información estratégica. Destacamos los éxitos clave del programa nacional para el control y tratamiento de la hepatitis C: establecimiento de la gobernanza y planificación nacional; desarrollo de capacidad diagnóstica; aprobación e introducción de tratamientos antivíricos de acción directa; formación de personal clave; generación de voluntad política y liderazgo; y promoción de asociaciones estratégicas clave. Los desafíos actuales y los próximos pasos del programa incluyen el desarrollo de un marco de seguimiento y evaluación detallado, así como herramientas para el seguimiento de la hepatitis viral. El gobierno necesita descentralizar todavía más la atención e integrar la gestión de la hepatitis C en los servicios clínicos corrientes para proporcionar un mejor acceso al diagnóstico y tratamiento de los pacientes. La introducción de pruebas diagnósticas rápidas en los centros de atención de salud pública ayudaría a aumentar la búsqueda de casos. El aumento de la financiación pública y privada es esencial para apoyar los servicios de atención y tratamiento.

## ملخص

أصبح علاج التهاب الكبد "سي" مجديًا بدرجة عالية ويمكن للمرضى تحمل آثاره، وذلك بفضل طرح الأدوية ذات التأثير المباشر المضادة للفيروسات. وبالرغم من ذلك، فإن استفادة المرضى من هذا العلاج لا تزال محدودة في البلدان منخفضة ومتوسطة الدخل، ويرجع ذلك إلى نقص المرافق الصحية الأساسية الداعمة وارتفاع تكلفة العلاج. وتشجع الهيئات الدولية البلدان الأكثر فقرًا على وضع أساليب للإغاثة في قطاع الصحة العامة من شأنها تيسير إتاحة الرعاية والعلاج على الصعيد الوطني. وبالرغم من ذلك، أجرى عدد قليل من البلدان توثيقًا لخطط وسياسات رسمية بهذا الشأن. وتتضمن هذه الوثيقة توضيحًا من جانبنا للنهج الذي اتبعته رواندا في إطار الصحة العامة بشأن السيطرة على التهاب الكبد "سي" وتوفير الرعاية اللازمة في ظل استراتيجية قطاع الصحة المعني بالتهاب الكبد والتابع لمنظمة الصحة العالمية. ويشمل هذا النهج وضع السياسات والبرامج اللازمة وتطبيقها، وبذل الجهود الرامية للوقاية من المرض، وتوفير المقدار اللازم من الفحص الطبي، وتقديم الخدمات العلاجية، واستخدام نظم للمعلومات الاستراتيجية. وإننا نسلط الضوء على التجارب الرئيسية الناجحة المتعلقة بالبرنامج الوطني للتعامل مع التهاب الكبد "سي" والسيطرة عليه: وضع سبل وطنية للإدارة والتخطيط؛ وتطوير القدرة على تشخيص المرض؛ واعتماد العلاجات ذات التأثير المباشر المضادة للفيروسات وطرحها للمرضى؛ وتدريب الموظفين الرئيسيين؛ وإيجاد الإرادة السياسية والقيادة اللازمة؛ وتعزيز الشراكات الاستراتيجية الهامة. وتشتمل التحديات القائمة حاليًا والخطوات التالية في البرنامج على وضع إطار مفصل للمراقبة والتقييم وإيجاد وسائل للرصد فيما يتعلق بالتهاب الكبد الفيروسي. وتحتاج الحكومة إلى مزيد من تحول الرعاية الصحية إلى اللامركزية ودمج التعامل مع التهاب الكبد "سي" داخل الخدمات العلاجية المعتادة لتحقيق قدر أكبر من استفادة المرضى من خدمات التشخيص والعلاج. ومن شأن توفير اختبارات التشخيص السريع في المنشآت العلاجية لتقديم الرعاية في مجال الصحة العامة أن يساعد في زيادة معدل اكتشاف الحالات المرضية. وتلعب الزيادة في التمويل الحكومي والخاص دورًا جوهريًا في دعم خدمات الرعاية الصحية والعلاج.

## 摘要

随着直接抗病毒药物的引入，丙型肝炎的治疗既高效又可耐受。然而，由于缺乏支持性的卫生基础设施和高昂的治疗费用，中低收入国家中的患者获得治疗的机会仍然有限。国际机构正在推动较贫穷的国家组织公共卫生对策，以促进全国范围内的护理和治疗。但是很少有国家记录正式的计划和政策。在这里，我们概述了卢旺达公共卫生框架在世界卫生组织肝炎健康部门战略范围内为控制丙肝和保健所采取的方法。这包括政策、方案、预防工作、筛查能力、治疗服务和战略信息系统的制定和执行。我们强调国家计划在丙型肝炎控制和管理上取得的关键成就：建立国家治理和规划；提高诊断能力；批准和引进直接抗病毒治疗；培训重要人员；产生政治意愿和领导能力并促进重要的战略伙伴关系。该计划面临的现有挑战和下一步措施包括制定监测病毒性肝炎的详细监测与评估框架和工具。政府需要进一步下放护理，将丙型肝炎管理纳入常规临床服务，为患者提供更好的诊断和治疗。公共医疗机构引进快速诊断测试将有助于增加病例发现。增加公共和私人融资对支持护理和治疗服务至关重要。

## Резюме

Благодаря введению противовирусных препаратов прямого действия лечение гепатита С стало одновременно высокоэффективным и переносимым. Однако доступ для пациентов к лечению остается ограниченным в странах с низким и средним уровнем доходов из-за отсутствия поддерживающей инфраструктуры здравоохранения и высокой стоимости лечения. Международные организации оказывают поддержку более бедным странам в организации мероприятий по реагированию в области общественного здравоохранения, которые могли бы способствовать внедрению ухода и лечения в национальном масштабе. Однако лишь немногие страны имеют официальные планы и политику. Здесь авторы описывают подход в области общественного здравоохранения, принятый в Руанде для контроля и лечения гепатита С в рамках стратегии сектора Всемирной организации здравоохранения по борьбе с вирусным гепатитом. Этот подход включает разработку и внедрение политики и программ, профилактических мероприятий, возможностей скринингового обследования, лечебных услуг и стратегических информационных систем. Освещаются ключевые успехи в рамках национальной программы по контролю и лечению гепатита С: создание национального управления и планирования, развитие диагностического потенциала, регистрация и внедрение противовирусных препаратов прямого действия, подготовка основного персонала, формирование политической воли и лидерства, а также укрепление ключевых стратегических партнерских отношений. Существующие проблемы и последующие шаги для этой программы включают разработку детальной системы мониторинга и оценки, а также инструментов мониторинга вирусного гепатита. Правительство должно децентрализовать медицинский уход и интегрировать лечение гепатита С в обычные медицинские учреждения, чтобы облегчить доступ для пациентов к диагностике и лечению. Внедрение экспресс-диагностических тестов в государственные медицинские учреждения поможет увеличить выявление случаев инфицирования. Увеличение государственного и частного финансирования имеет важное значение для поддержки системы здравоохранения и медицинского обслуживания.

## Introduction

Worldwide, an estimated 71 million people, 1% of the global population, are chronically infected with hepatitis C virus (HCV).^1^ The prevalence in sub-Saharan countries is significantly higher. In a meta-analysis of 1 151 337 individuals it was estimated to be 2.7% overall, with regional variations.^2^ The introduction of direct-acting antiviral drugs have led to simplified treatment of hepatitis C and promising outcomes. Yet, the number of deaths from the disease is on the rise globally.^3^ For many low- to middle-income countries, the high cost of medication, alongside limited laboratory and health-system capacity, prevents many of them from accessing and rolling out treatment on a national scale.^4^ Voluntary licence agreements with manufacturers have allowed 101 countries, including Rwanda, to receive direct-acting antivirals at reduced prices. Nevertheless, the cost is still prohibitive for most patients in low- and middle-income countries.^5^^,^^6^ Due to the rapid advances in hepatitis C treatment, global bodies, such as the World Health Organization (WHO), World Hepatitis Alliance and the Centers for Disease Control and Prevention, are encouraging low- and middle-income countries to develop organized national responses to the disease.^7^ However, to date, few reports describe the process of planning or implementing responses to hepatitis C disease at the country level. Although 43 countries have reported completion of national viral hepatitis elimination plans, very few of these are from low- and middle-income countries and even fewer are from sub-Saharan Africa.^1^

Rwanda, a low-income country as classified by the World Bank, has well-established achievements in maternal and child health and control of human immunodeficiency virus (HIV), tuberculosis and malaria. It was also one of the few countries in sub-Saharan Africa to achieve most of the health-related millennium development goals.^8^ Rwanda has now increased its attention to the hepatitis C epidemic.^9^ We describe the framework for a comprehensive response to the epidemic in Rwanda within the proposed framework of the WHO hepatitis health sector strategy.^7^ We focus on five areas: (i) information for focused action, (ii) interventions for impact, (iii) delivering for equity, (iv) financing for sustainability and (v) innovation for acceleration. We highlight several accomplishments, as well as challenges, within these strategic areas. These may inform the planning and implementation of national hepatitis C control measures by other low- and middle-income countries, as well as the way forward to control the disease successfully within Rwanda.

## Information for focused action

The seroprevalence of HCV antibody has most recently been estimated to be 3.1%^10^ within the general Rwandan population of 11.9 million people^11^ and 4.7% (5470)^12^ among the 116 868 people living with HIV. Whereas hepatitis C prevention efforts have been increasing, we found no existing surveys or qualitative data regarding attitudes, knowledge or beliefs towards viral hepatitis in the general population in Rwanda, reflecting an overall lack of published literature on this topic in sub-Saharan Africa. In addition, the causes of HCV transmission in Rwanda are largely unknown, but are assumed to include past traditional medical practices, unsafe injection practices during and before the 1970s, exposure to contaminated blood during the Rwandan genocide of 1994 and blood transfusions before 1999.

In 2011, the Government of Rwanda established its hepatitis control unit under the Division of HIV/AIDS, STIs and Other Blood Borne Infections at the Rwanda Biomedical Centre. The objective was to develop a specific programme for the prevention, care and treatment of hepatitis B and C.^13^ In 2013, the viral hepatitis technical working group was set up, comprising health ministry specialists, clinicians, academic researchers, laboratory experts, implementing partner organizations, United Nations agencies, and civil society and private sector representatives. The group began to meet regularly to provide technical advice for the design and implementation of the programme. At that time, a national policy on viral hepatitis prevention and management in Rwanda was developed and published to provide specific guidance to health-care providers and facilities on the implementation of the clinical guidelines.

In April 2016, a patient chart for hepatitis C care and treatment was developed and implemented in paper form at hospitals receiving HCV patients. This was to support clinical decision-making according to national guidelines and allow proper reporting of patient outcomes. The group also developed patient registers that were implemented to track the overall number of patients enrolled in clinical and laboratory services. As of October 2017, Rwanda Biomedical Centre started developing and reviewing national indicators for hepatitis C to be included in the integrated health management information system in Rwanda.

## Interventions for impact

Rwanda began a systematic blood donation screening programme for HIV, HBV and syphilis in 1985, and introduced screening for HCV in 1999.^14^ The 1994 genocide left the country’s blood safety programme in disarray and may have fuelled direct hepatitis transmissions via mass casualties and injuries. Repeated rape and penetrating trauma under torture exposed thousands of survivors to potential contact with HIV or HCV.^15^

The capacity for HCV testing for patients was first introduced in Rwanda in 2013 and was initially recommended for high-risk groups, including patients with HIV or HBV infection, intravenous drug users, infants of mothers with hepatitis C, individuals who received blood or organ transplants before 1999, individuals with occupational exposure and other patients as clinically indicated.^16^ National guidelines recommend one-time testing for all adults, followed by annual follow-up testing for cases of potential exposure. Higher-risk groups, such as intravenous and intranasal drug users and female sex workers, as well as health-care workers and community health workers, are recommended to undergo annual testing. Systematic testing of pregnant women is also recommended.^17^ Currently, targeted screening campaigns are underway for adults older than 45 years, health-care workers and laboratory technicians, men who have sex with men, commercial sex workers and prisoners. Of note, injection drug use is rare in Rwanda and is not considered a primary driver of bloodborne epidemics. The health ministry has conducted routine general training and education for health practitioners about risk reduction for iatrogenic exposure to hepatitis, including reduction of unnecessary injections and increased infection control measures at health facilities.

HCV testing guidelines in Rwanda include antibody testing using enzyme-linked immunosorbent assay (ELISA) or a rapid diagnostic test, which is then confirmed with polymerase chain reaction-based viral load quantification. Currently, 11 hospitals nationwide have the capacity to perform ELISA-based testing for HCV. Although several rapid diagnostic tests have been validated in Rwanda, there is no consensus on product selection or procurement, and rapid diagnostic tests are not currently available in public health-care facilities. As part of the well-established health services for HIV, 10 hospitals have the capacity to conduct HCV viral load testing. Using current systems for HIV viral load testing, blood samples for HCV testing are collected at local health centres and district hospitals and delivered to one of the testing sites via a centrally organized transport system, which also delivers results back to the health facilities. Given the predominance of HCV genotypes 1 and 4 in Rwanda,^18^ genotyping is not currently recommended before starting patients on direct-acting antiviral drugs according to Rwanda’s guidelines. In 2016, the health ministry conducted a nationwide testing campaign for both HBV and HCV in over 150 000 adults living with HIV.

In 2013, the health ministry developed the first national guidelines for hepatitis prevention and treatment, which included guidance on screening, diagnosis and treatment for both hepatitis B and C within the Rwandan context.^19^ The guidelines were updated in June 2015 to include detailed guidance for treatment with direct-acting antiviral drugs and avoidance of interferon-based therapy for treatment-naïve patients.^17^ The guidelines recommended sofosbuvir (400 mg) with ribavirin (1000–1200 mg) for 24 weeks; sofosbuvir (400 mg) with ledipasvir (90 mg) for 12 weeks; or sofosbuvir (400 mg) with daclatasvir (60 mg) for 12 weeks. The choice of regimen depends on clinical indications, as well as availability and affordability of the drugs to the patient. The guidelines recommended that, whereas all patients with chronic infection should be considered potential candidates for therapy, priority should be given to patients with advanced fibrosis (aspartate aminotransferase to platelet ratio index > 1.0, or symptoms of liver cirrhosis) and for patients co-infected with HIV subtype 1 who are at-risk of accelerated disease progression.

In August 2015, the health ministry officially authorized the use of sofosbuvir or sofosbuvir with ledipasvir for treatment of hepatitis C in Rwanda. The government was given preferred pricing for these drugs via licensed manufacturers at a cost per 28 days of 300 United States dollars (US$) for sofosbuvir and US$ 400 for sofosbuvir with ledipasvir in 2015 (subsequently reduced to US$ 260 in 2017). A donation of daclatasvir (2000 treatment regimens) was also obtained through the Clinton Health Access Initiative Quick-Start programme. As of June 2017, over 2500 of an estimated 55 000 patients requiring medication^20^ have been treated with direct-acting antiviral drugs, and treatment for 9000 additional patients has been procured via support from The Global Fund to Fight AIDS, Tuberculosis and Malaria. Medications are dispensed by three dispensaries in the capital city Kigali, licensed by the distributor: two at treatment referral centres and one at a centrally located private pharmacy. The hepatitis C national treatment selection committee, composed of senior clinicians appointed by the health ministry in February 2016, reviews all medical prescriptions. There are now 20 physicians approved to prescribe direct-acting antivirals at 10 districts, provincial and referral hospitals which provide treatment coverage for all five provinces in Rwanda. The plan for decentralization and scale-up of hepatitis C treatment includes the establishment of treatment capacity at all 48 district hospitals countrywide by 2020. However, this initiative is limited by diagnostic capacity, the availability of trained personnel and financial barriers to diagnostics and treatment.

## Delivering for equity

The health ministry has introduced mass-media campaigns to raise awareness and reduce the general stigma surrounding hepatitis C. The *New Times*, a popular newspaper printed in English, published 29 articles about viral hepatitis from 2009–2015, with nine of those articles published in 2015. In 2015, Rwanda officially first commemorated World Hepatitis Day with a public walk advocating for hepatitis prevention and treatment.^21^ The health ministry and Rwanda Biomedical Centre have committed to conducting an annual public campaign to raise awareness through community health workers and radio and television, to provide mass screening information and to link patients to care. The Rwanda Organization for the Fight Against Hepatitis was established in 2013 and has focused on providing education on hepatitis to the general population and advocating for availability and access of new treatment modalities in Rwanda.

The government has promoted the decentralization of health services to provide a more comprehensive package of health services at provincial hospitals, district hospitals, health centres and at the community level through community health workers. This process was pioneered through the response to the HIV epidemic. The HIV response resulted in 524 facilities providing antiretroviral therapy and 1068 physicians and nurses with the capacity to initiate and maintain patients on therapy.^22^ This network now serves as a platform for care delivery for other chronic infections (e.g. hepatitis B virus infection) and noncommunicable diseases (e.g. hypertension and diabetes). Since 2015, the health ministry, in collaboration with Duke University, provided 1-week advanced training in hepatitis C disease care and treatment to 20 internal medicine specialists, 72 general physicians and 48 specialist nurse mentors. General 5-day trainings on hepatitis C care and treatment were provided by Rwanda Biomedical Centre for 550 nurses, 24 laboratory technicians, 48 hospital pharmacists, 48 nutritionists and 47 data managers working in HIV services at both the district hospital and health centre level in all 30 districts. Clinical mentorship for viral hepatitis was integrated into the established clinical mentorship programme for HIV. This included routine visits by expert clinicians for theoretical and practical clinical teaching of physicians and nurses at health centres, after their general training in hepatitis. Despite these efforts, there remains a bias towards diagnosis and treatment of patients at urban referral hospitals, and care is largely limited to patients who are able to purchase diagnostics and treatments out of pocket or who are covered through various health insurance schemes.

## Financing for sustainability

In October 2016, the technical working group developed and approved the Rwanda viral hepatitis national operational plan. The plan demonstrated priority-setting of key national activities and provided cost estimations for different levels of coverage of screening, diagnosis, and treatment of both hepatitis B and C. The annual cost of a comprehensive national viral hepatitis programme was estimated at US$ 168.6 million.^20^ Currently, all major private health insurance companies in Rwanda, as well as the military medical insurance, reimburse for the cost of both sofosbuvir with ribavirin and sofosbuvir with ledipasvir. Additionally, the Rwanda social security board, which provides health insurance for public servants, covers 85% of the cost of these drugs for patients. These sources in combination provide coverage to an estimated 7.3% (730/9998) of insured households in Rwanda,^23^ comprising most of the patients started on treatment to date. Reimbursement for HCV viral load testing and hepatitis C treatment by the community-based health insurance scheme, which provides health insurance coverage to at least one member of 97.1% (9708/9998) of households,^23^ is currently under discussion.

As part of the national operational plan, several different costing scenarios were calculated for the prevention, diagnosis and care of hepatitis B and C. In a full intervention scenario ‒ where all screening, diagnosis and treatment options are implemented ‒ the cost for HCV screening of 2.6 million people and treatment of 74 105 people annually was projected to be approximately US$ 128 million. If treatment were reserved for patients with HIV coinfection or advanced disease (aspartate aminotransferase to platelet ratio index > 1.0), then the total annual cost to implement the hepatitis C control programme was projected to be US$ 52 million. In comparison, the estimated annual expenditure for HIV care in Rwanda from 2013‒2018 is around US$ 207 million.^20^

Financing for the hepatitis C response to date has largely been through expenditure by the government and there has been a strong commitment from the highest levels of the government to funding hepatitis-C-related programming, operations and consumables.^24^ This has resulted in a doubling of the budget earmarked for viral hepatitis from 2013 to 2016 and expanded targets in the forthcoming Rwanda health sector strategic plan 2018–2023. Further reductions in the price of generic pharmaceuticals, projected to reach US$ 100 per month (or US$ 300 per treatment) are anticipated to result in greater affordability of drugs. This would facilitate hepatitis C therapy for uninsured patients and those in the community-based insurance programme, which provides the financial coverage for care services for the majority of the Rwandan population. Irrespective of this development, financing for the hepatitis C response via both domestic and external mechanisms remains a major barrier in Rwanda as it has for other low- and middle-income countries globally.^25^

## Innovation for acceleration

Several key elements have accelerated Rwanda’s success in building a comprehensive framework for the public health response to hepatitis C with resource constrains. Hepatitis C screening, diagnosis and treatment have been integrated into existing programmes and systems for HIV infection. These systems cover policy and guidelines development, laboratory equipment, specimen transport, clinic infrastructure, training platforms, medication supply chain and clinical training and mentorship. This strategy has allowed for rapid implementation of the hepatitis C response. Although a plan for rapid decentralization exists, challenges remain in training adequate numbers of health-care personnel and equipping laboratories to provide maximal access and equitable distribution of services. The involvement of community and hepatitis C patient networks, particularly in advocacy for approval and access to direct-acting antivirals, has been essential in Rwanda’s push to rapidly introduce new diagnostics and therapies. Such networks will continue to play an important role in public awareness efforts. Finally, under the technical working group framework and building on HIV control experiences, partnerships were established between the health ministry and several international organizations, including Stanford University, Maryland University, Duke University, Gilead Sciences, the Clinton Health Access Initiative, Partners In Health, the Centers for Disease Control and Prevention and WHO. These partnerships leverage technical, financial and operational expertise and experience with hepatitis C. The health ministry has been active in building the key partnerships required to develop a rapid, informed and sustainable approach to a national hepatitis C programme. The ministry involved international academic institutions in training and research, insurance companies in the financing of diagnostic testing and medications, pharmaceutical companies in rapid price reductions and procuring direct-acting antivirals, and nongovernmental organizations in securing donations and informing planning and policy.

## Conclusions

In Rwanda, a robust programme for hepatitis C control has been developed, including key accomplishments in the areas of establishing national governance and planning, developing diagnostic capacity, approving the use of direct-acting antiviral treatment, training key personnel, generating political will and leadership from within the government and fostering key strategic partnerships for technical support (Table 1). However, major challenges remain, with key next steps to be undertaken. The government will need to focus on developing a detailed monitoring and evaluation framework and tools for monitoring of viral hepatitis. Care needs to be further decentralized from referral and provincial hospitals to the district hospital level, with hepatitis C management integrated into routine clinical services to provide more equitable access to diagnosis and treatment. Procurement and distribution of rapid diagnostic tests are needed to increase coverage of diagnostic capacity and to facilitate easier and more efficient diagnosis of hepatitis C. Most importantly, there remain financial constraints on the care and treatment needed to control the epidemic effectively and limit direct out-of-pocket spending by the population. The government will need to increase both public sector investment as well as support from private and external funding sources. The successes and challenges learnt from Rwanda’s response to hepatitis C will be valuable as elimination efforts are developed and accelerated in low- and middle-income countries around the world.

**Table 1:** World Health Organization hepatitis health sector strategy and Rwanda response to hepatitis C

WHO			Rwanda	
Strategic area^a^	Major areas^a^	Actions		Next steps
Information for focused action	Information for action	Viral hepatitis indicators established and elaborated. All patients on treatment monitored using standardized charts.		Incorporate indicators into national health management information systems platform. Develop detailed national monitoring and evaluation framework and tools. Design and collect data from epidemiology surveys.
	National plans	Health ministry hepatitis control unit established. National viral hepatitis policy and national strategic plan for viral hepatitis developed and published. National viral hepatitis technical working group and national hepatitis C treatment selection committee established and functional. Viral hepatitis targets incorporated into HIV national strategic plan 2013–2020.		Viral hepatitis guidelines to be updated in 2018.
Interventions for impact	Blood safety	Systematic screening of blood donors (since 1999). Laboratory quality control measures established.		Link blood donors who screen positive to care via a reference system.
	Infection control	Injection safety policy and infection control guidelines available in all health facilities.		Implement infection control at community level through monitoring unsafe nonmedical and traditional practices.
	Safer sex	Programmes offering distribution of free condoms well established via HIV national strategic plan.		None planned.
	Diagnosing infection	HCV testing recommended for target populations in 2013 and universal testing in 2015. HCV antibody testing by ELISA available at 11 hospitals and PCR assay available at 10 hospitals. All people living with HIV/AIDS screened for HCV. Screening campaigns underway for high-risk groups.		Procure and distribute rapid diagnostic testing for all health centres. Ensure PCR testing capacity for all district hospitals.
	Treatment and care	Clinical guidelines and policies for care and treatment available at all facilities. Direct-acting antivirals established as standard of care, approved for use and available on market. Twenty health-care providers licensed at 10 treatment centres countrywide.		Increase number of treatment centres to include all district hospitals.
Delivering for equity	Adapting services	National coverage for referral network for hepatitis C treatment achieved. Screening and testing done at hospitals from multiple entry points (including antenatal care, HIV centres, outpatient departments and blood transfusion centres). Individuals testing positive linked to care. All health centres and hospitals have at least one health-care provider trained on management of viral hepatitis.		Educate community health workers on basic knowledge of viral hepatitis prevention. Establish quality improvement and monitoring of hepatitis services. Strengthen linkages with blood transfusion centres and maternal and child health, noncommunicable diseases, and mental health services. Develop and implement district operational plans.
	Human resources	Training for physicians, nurses, laboratory technicians, pharmacists and nutritionists conducted in all 30 districts. Hepatitis C integrated into clinical mentorship programme on infectious diseases for health-centre nurses. Routine training for health-care personnel on infection control.		Implement continuing education on hepatitis C for nurses and physicians nationwide. Integrate management of hepatitis into formal teaching curricula.
	Access to medicines, diagnostics, and other commodities	Centralized procurement of hepatitis C diagnostics and medication via the ministry of health.		Obtain further hepatitis C medication price reductions from manufacturers of generic drugs.
	Enabling environment	Political commitment to support viral hepatitis programme strengthened. Rwanda Organization for Fighting Against Hepatitis. World Hepatitis Day, media attention, advocacy groups and outreach campaigns established. Youth education on viral hepatitis prevention by health professionals. Access to viral hepatitis services ensured, regardless of sex.		Develop broader hepatitis C messaging and mass-media campaigns. Call for action to private sector and external funders to invest in viral hepatitis programme.
Financing for sustainability	Financing for sustainability	National operational plan implemented, with costing of diagnosis and treatment at various access levels. Current diagnostics and treatment obtained at discount pricing. Various public and private insurers cover up to 85% of hepatitis C diagnostic and treatment costs.		Further elaborate a detailed case for investment by government in viral hepatitis control. Community-based health insurance to provide coverage for hepatitis C-related costs. Understand and monitor government health expenditures related to hepatitis C care and treatment.

AIDS: acquired immune deficiency syndrome; ELISA: enzyme-linked immunosorbent assay; HCV: hepatitis C virus; HIV: human immunodeficiency virus; PCR: polymerase chain reaction; WHO: World Health Organization.

^a^ Based on the WHO global health sector strategy on viral hepatitis 2016–2021.^7^
